# Unusual Presentation of Polyautoimmunity and Renal Tubular Acidosis in an Adolescent With Hashimoto's Thyroiditis and Central Pontine Myelinolysis

**DOI:** 10.3389/fendo.2020.548877

**Published:** 2020-10-14

**Authors:** Nora Bruns, Ilja Finkelberg, Ibrahim Al-Attrach, Peter F. Hoyer, Rainer Büscher

**Affiliations:** ^1^Department of Pediatrics I, Neonatology, Pediatric Intensive Care, Pediatric Neurology, University Hospital Essen, University Duisburg-Essen, Duisburg, Germany; ^2^Department of Pediatrics II, Pediatric Nephrology, University Hospital Essen, University Duisburg-Essen, Duisburg, Germany

**Keywords:** case report, autoimmune thyroiditis, distal renal tubular acidosis, central pontine myelinolysis, Hashimoto's thyroiditis, Sjögren's syndrome, celiac disease, hypokalemia

## Abstract

**Background:** Hashimoto's thyroiditis is frequently associated with other autoimmune diseases and may include renal involvement.

**Case description:** A 17-year-old female with previously diagnosed Hashimoto's thyroiditis and vitiligo was admitted to a pediatric intensive care unit with hypokalemic paralysis and acidosis, after having suffered from recurrent muscular weakness for approximately one year. A few days later she developed central pontine myelinolysis. After initial stabilization she was also diagnosed with distal renal tubular acidosis (dRTA) and tubular proteinuria which can occur in Sjögren's syndrome. Extended screening for autoimmune diseases additionally revealed celiac disease. Treatment with Prednisone and substitution of potassium quickly lead to the resolution of proteinuria and dRTA, but unilateral paralysis of the sixth nerve as a result of central pontine myelinolysis was irreversible.

**Conclusions:** This is the rare case of polyautoimmunity including autoimmune thyroiditis, Sjögren's syndrome, vitiligo and celiac disease in an adolescent with few disease-specific symptoms. The diagnoses were made via a complicating nephritis causing dRTA and proteinuria. Delay in diagnosis lead to permanent neurological damage. This case highlights the need for pediatricians to be aware of rare accompanying diseases and their complications in “common” pediatric autoimmune diseases like Hashimoto's thyroiditis and celiac disease.

## Introduction

Hashimoto's thyroiditis is the most common autoimmune thyroiditis in children and adolescents and frequently associated with other autoimmune diseases including vitiligo, rheumatoid arthritis, polymyalgia rheumatica, celiac disease, diabetes, and Sjögren's syndrome (1, 2). The presence of more than one well-defined autoimmune disease in one patient is called polyautoimmunity (3). Various autoimmune disorders can affect the kidneys leading to interstitial nephritis with tubular dysfunction that results in electrolyte loss (distal renal tubular acidosis, dRTA). These autoimmune diseases include Hashimoto's thyroiditis, systemic lupus erythematosus and Sjögren's syndrome (4–7). Celiac disease has also been linked to kidney disease (8, 9). dRTA leads to hypokalemia that can be complicated by hypokalemic paralysis. As a matter of fact, hypokalemic paralysis can be the first manifestation of Sjögren's syndrome (10, 11). A possible complication of hypokalemia of different origins is central pontine myelinolysis, which has repeatedly been reported in the context autoimmune disorders and dRTA (12–15).

Here, we report the case of a female adolescent with Hashimoto's thyroiditis whose polyautoimmunity was unveiled via renal disease leading to severe neurological complications. Two years after being diagnosed with euthyroid Hashimoto's thyroiditis, the girl developed recurrent episodes of muscular weakness and pain that were associated with metabolic acidosis and hypokalemia. Only after developing severe hypokalemia with consecutive central pontine myelinolysis, she was diagnosed with polyautoimmunity that caused interstitial nephritis and dRTA.

## Case Description

A 17-year-old girl presented to the emergency room with muscular weakness and leg pain of ~2 days duration. For one year she had repeatedly suffered from muscular weakness associated with hypokalemia and metabolic acidosis. Because of the weakness and joint pains, she had presented to doctors of several pediatric subspecialties including neurology, rheumatology, cardiology, and nephrology. Periodic hypokalemic paralysis was primarily suspected, whereas the accompanying acidosis and elevated antinuclear antibody (ANA) titers (1:2560 [<1:80]) could not be explained then. Three years before the present admission, autoimmune thyroiditis had been diagnosed but required no medical treatment.

**Figure 1:**
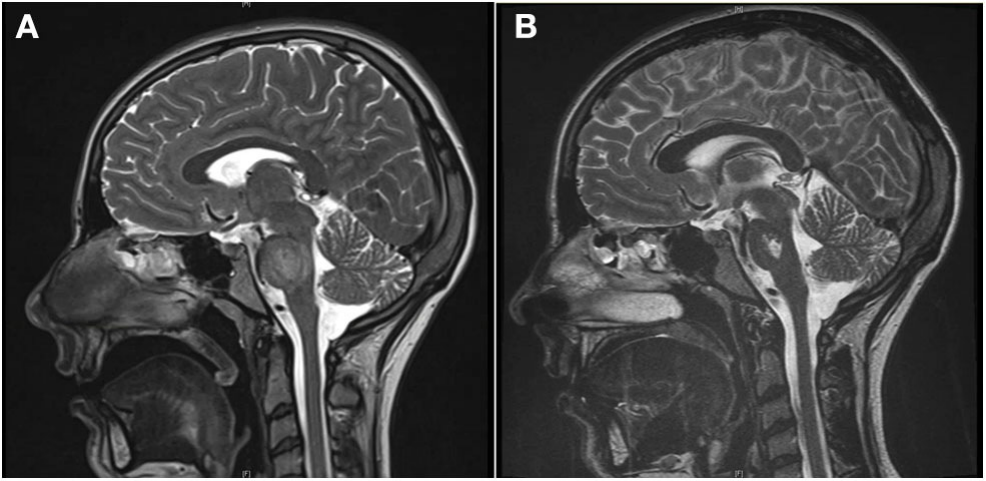
**(A)** The T2-weighted magnetic resonance tomography shows a diffuse increase of signal intensity and edema in the pons during the acute phase of central pontine myelinolysis. **(B)** Four weeks later, the edema has resolved, but a trident-like substance defect in the pons remains (T2 weighting).

Upon admission, she presented with severe hypokalemia (Potassium 1.8 mmol/l) and severe hyperchloremic acidosis (standard bicarbonate 13.3 mmol/l, Chloride 127 mmol/l) with normal anion gap. She was admitted to a pediatric intensive care unit and intravenous potassium substitution was performed with up to 6 mmol/kg/day. Five days after admission, the patient began to suffer from double images, impairment of speech, and general retardation. Magnetic resonance tomography revealed central pontine myelinolysis (Figure 1).

She was transferred to the pediatric nephrology department for further diagnostics and treatment. Upon admission, the patient was in stable condition and fully oriented. Neurological symptoms were bilateral palsy of the sixth nerve, dizziness, and slurred speech. She was unable to sit for more than half a minute, stand, or walk, whereas sensitivity and muscle power were not affected. Vitiligo could be observed on knees, chest, and upper back. Serum electrolytes and blood gas analysis were unremarkable.

**Figure 2:**
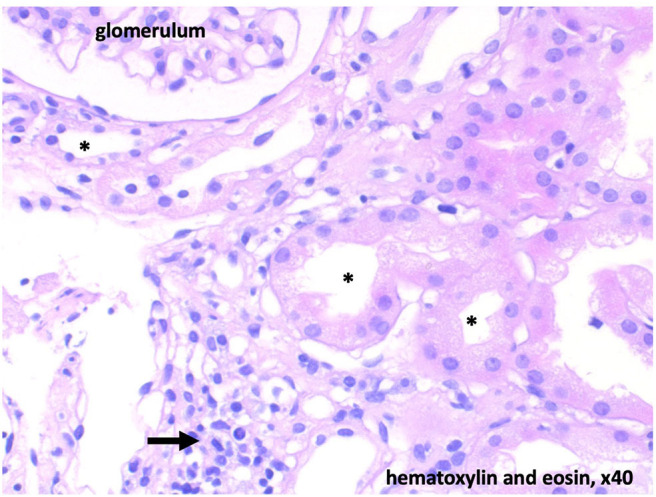
Histology with low-grade chronic and floride tubulointerstitial nephritis. Diffuse interstitial infiltrates (arrow). Acute tubulus damage is light to moderate and potentially reversible. * tubular lumen.

Due to the persistent metabolic acidosis and high potassium demand, we screened for renal disease. Renin was normal and Aldosterone low. The blood pressure ranged between the 5th and 50th percentile. Alkaline urine (pH 7.0), increased fractional potassium excretion (>20 %), and tubular proteinuria pointed toward tubular dysfunction. Due to the accompanying severe hypokalemia and metabolic acidosis (with normal anion gap), we suspected distal renal tubular acidosis (dRTA). Acquired dRTA frequently results from nephritis due to autoimmune disorders, e.g. systemic lupus erythematodes and Sjögren's syndrome. Supporting these differential diagnoses, central pontine myelinolysis can occur in both conditions. The consecutive renal biopsy revealed chronic and acute tubulointerstitial nephritis (Figure 2). Serum markers for Sjögren's syndrome (anti-Ro, anti-La, ANA) were strongly positive, whereas antibodies for systemic lupus erythematodes were negative. Extended screening for autoimmune diseases confirmed Hashimoto's thyroiditis and newly revealed celiac disease, which was verified by biopsy. Even after receiving the diagnosis of celiac disease, our patient denied having any gastrointestinal symptoms. Schirmer's test, ultrasound of the parotid gland and repeated extensive anamnesis could not reveal sicca symptoms. No signs of peripheral neuropathy or central nervous system involvement other than a persistent substance defect caused by central pontine myelinolysis could be found (Figure 1).

**Figure 3:**
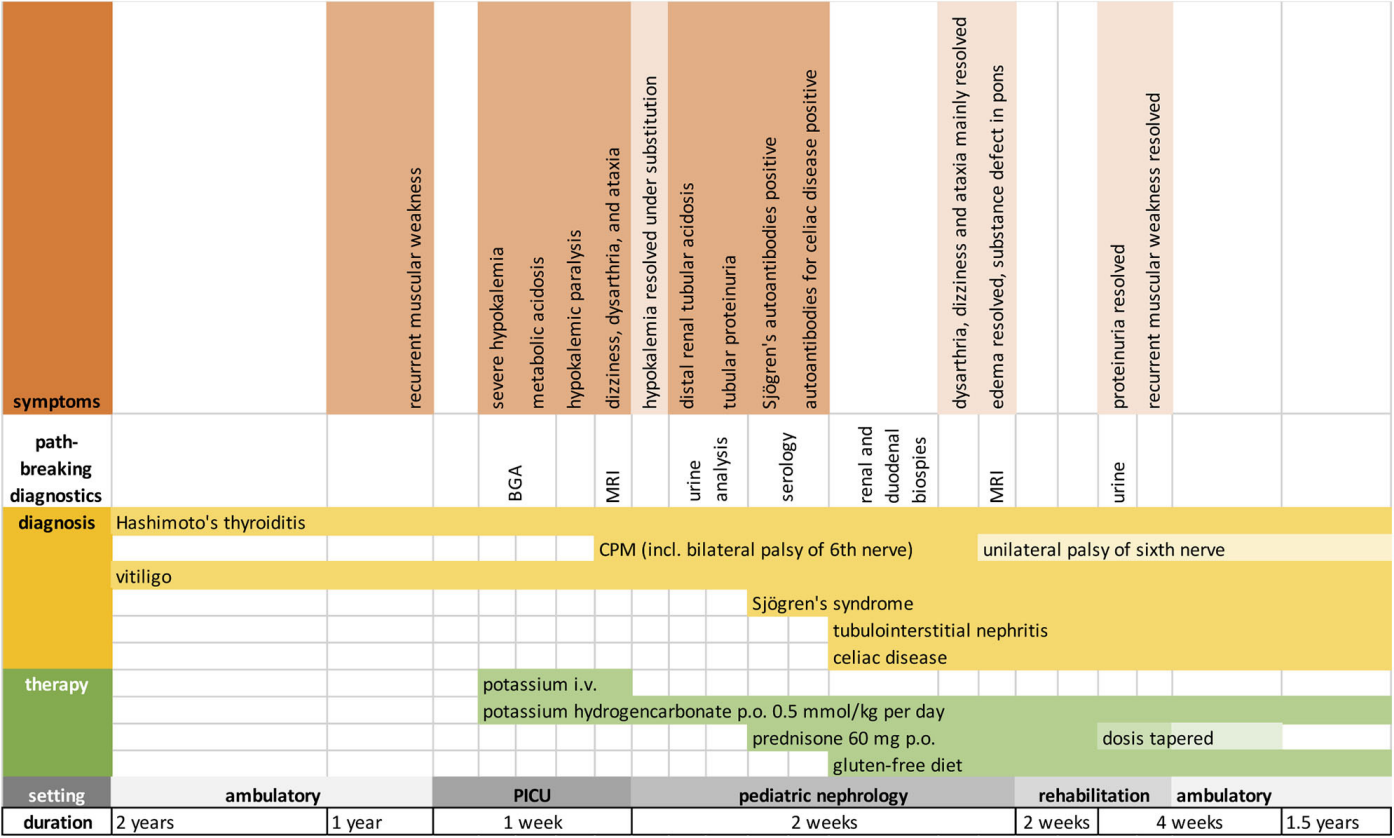
Timeline showing the chronological order of symptoms, diagnoses, and treatment. BGA, blood gas analysis; MRI, magnetic resonance imaging.

During the hospital stay, oral substitution of potassium hydrogencarbonate (0.5 mmol/kg potassium/day) stabilized electrolyte and metabolic homeostasis. Causal immunosuppressive treatment was initiated with oral Prednisone 60 mg/d and gluten-free diet. Additionally, intensive physiotherapy was initiated. Our patient quickly learned to cope with her neurological deficits that were partially reversible. However, unilateral palsy of the sixth nerve remained, accompanied by double images, intermittent dizziness, and perceived weakness upon physical activity. Four weeks after initiation of treatment, proteinuria was no longer detectable, and prednisone was tapered over the course of 2 months. Substitution of potassium hydrogencarbonate continues and maintains serum electrolytes and standard bicarbonate within normal range to date. Figure 3 depicts important symptoms, diagnostics, and treatment in a timeline.

## Discussion

We describe the unusual case of a patient with polyautoimmunity formed by Hashimoto's disease, celiac disease, vitiligo, and Sjögren's syndrome. Especially autoimmune thyroiditis and Sjögren's syndrome are often associated with polyautoimmunity (16). In our patient, clinical symptoms of the autoimmune diseases were mild or did not exist. This may partially explain the delay in diagnosis that made severe hypokalemia with consecutive central pontine myelinolysis and persisting neurological sequelae possible. Sjögren's syndrome was diagnosed via the complicating nephritis, which results from peritubular infiltration of lymphatic cells and leads to tubular dysfunction. Urine analysis to detect this dysfunction, represented by tubular proteinuria and urine potassium loss (dRTA) gave the crucial cue, as dRTA is frequently caused by Sjögren's syndrome in adults. However, in children Sjögren's syndrome is very rare. Adult population prevalence differs between countries and lies between 0,09 and 1,6% in western European countries—data for children and adolescents are lacking, but believed to be lower (17–20). Recurrent Parotitis is the most common symptom in children with Sjögren's syndrome, whereas adults normally present with sicca symptoms (21, 22). To make diagnosis even more difficult, it is very rare that renal complications are the leading symptoms of Sjögren's syndrome among children. We found only one case report with hypokalemic paralysis revealing Sjögren's syndrome in a 16-year old girl (23).

Hashimoto's thyroiditis was the only pre-existing condition that had been diagnosed, when our patient started consulting doctors about one year before she became critically ill. Autoimmune thyroiditis is frequently associated with other autoimmune disorders that show different clustering depending on age at diagnosis (24). Concomitant autoimmune diseases in children are typically type 1 diabetes and celiac disease, whereas adults are more likely to suffer from arthropathies and connective tissue diseases (24). Thus, our patient matches the “pediatric cluster” even though she did not suffer from gastrointestinal symptoms. This fits with the observation that ~50% of celiac disease is diagnosed in adulthood or adolescence and symptoms in the majority of patients are subtle (2). The most common accompanying autoimmune skin disease in autoimmune thyroiditis in all age groups is vitiligo, which was also present in our patient (24). The coexistence of autoimmune thyroiditis and Sjögren's syndrome was examined by various studies and is attributed to shared pathophysiological mechanisms (25–28). Because of their common genetic and pathophysiological background, it has been suggested that patients with autoimmune thyroiditis who remain unwell despite treatment or develop new unspecific symptoms should be screened for accompanying autoimmune disorders (1).

Initially, our patient did not receive treatment for autoimmune thyroiditis and was unwell for a long period of time. She already presented two autoimmune diseases—Hashimoto's thyroiditis and vitiligo—when she started consulting doctors because of her recurrent weakness. The specialists screened for further autoimmune diseases: e.g., systemic lupus erythematodes was excluded three times and a Schirmer's test (with negative result) was performed months before admission to hospital. Relevant health information was only collected by the family physician or pediatrician in a paper file, making it very difficult to unveil the complexity of this case. The diagnostic approach to generate the suspect diagnosis (urine analysis of proteins and electrolytes and literature search) was simple—concordantly, the treatment required to balance electrolytes and immunosuppression to induce “remission” was mild. This makes it even more tragic that our patient suffered from persistent neurological damage that could have been prevented by timely diagnosis and adequate treatment (immunosuppression to induce remission and substitution of potassium and bicarbonate).

In the future, increased awareness among pediatricians regarding polyautoimmunity and its comorbidities in pediatric autoimmune diseases (e.g., autoimmune thyroiditis, celiac disease, type 1 diabetes, Addison's disease, or atrophic gastritis) may possibly help to detect similar cases earlier. Although the combination and severity of our patient's diseases is extremely rare, pediatricians should be aware of rare diseases and their even more rare complications. A central, national or international database integrating relevant diagnostic information may contribute to a better understanding of complex and rare diseases.

## Ethics Statement

Written informed consent was obtained from the individual(s) for the publication of any potentially identifiable images or data included in this article.

## Author Contributions

All authors were involved in the patient's treatment. NB wrote the manuscript and designed figures, IF procured informed consent, follow-up information, and material for figures. IA-A, PH, and RB critically read and redacted the manuscript.

## Conflict of Interest

The authors declare that the research was conducted in the absence of any commercial or financial relationships that could be construed as a potential conflict of interest.
